# Nomogram Personalizes and Visualizes the Overall Survival of Patients with Triple-Negative Breast Cancer Based on the Immune Genome

**DOI:** 10.1155/2020/4029062

**Published:** 2020-11-24

**Authors:** Peipei Wang, Yang Fu, Yueyun Chen, Qing Li, Ye Hong, Ting Liu, Zhenyu Ding

**Affiliations:** Department of Biotherapy, Cancer Center, West China Hospital, West China Medical School, State Key Laboratory of Biotherapy, Sichuan University, Chengdu, China

## Abstract

**Background:**

Triple-negative breast cancer (TNBC) is usually poorly differentiated, highly invasive, susceptible to distant metastasis, and less responsive to endocrine and targeted therapy. However, immunotherapy is a promising treatment for TNBC patients recently.

**Methods:**

The prognostic value of immune-related genes (IRGs) was explored by using RNA sequencing and microarray data of 123 and 107 TNBC patients from TCGA and GEO databases, respectively.

**Results:**

In TCGA database, GO and KEGG pathway analysis of 119 differential IRGs indicated that they actively participate in the interaction of cytokines and receptors. A nomogram model constructed by the prognosis-related CCL25, IL29, TDGF3, GPR44, and GREM2 in the IRGs could personalize and visualize the 1-, 2-, 3-, 4-, and 5-year overall survival (OS) of TNBC patients. Moreover, TNBC patients could be defined as low-risk (risk score < 194) or high-risk (risk score ≥ 194) cohorts based on the risk score derived from the nomogram model. The results could be validated by the GSE58812 dataset. Furthermore, the risk score was an independent risk factor for TNBC patients (HR = 1.019, 95% CI 1.012-1.027, *p* < 0.001) and was positively related to stage (*p* = 0.017). Interestingly, the risk score could reflect the infiltration of B cells, CD4+ T cells, CD8+ T cells, dendritic cells, and neutrophils.

**Conclusion:**

These findings provided a reference for personalized OS prediction in TNBC patients and might be potential immune biomarkers for designing novel therapy.

## 1. Introduction

Breast cancer has high incidence in women. Different types of breast cancer have obvious differences in morphology, molecular pathological characteristics, clinical features, and responses to tumor treatment [1]. As a special subtype of breast cancer, triple-negative breast cancer (TNBC) lacks the expression of estrogen receptor (ER), progesterone receptor (PR), and epidermal growth factor receptor 2 (HER2), which severely limits the clinical usage of endocrine and targeted therapy.

TNBC is usually poorly differentiated, highly invasive, susceptible to distant metastasis, and less responsive to treatment than other hormone receptor-positive breast cancers, so it has a higher risk of early relapse [2]. However, due to its unstable genome and high mutation rate, TNBC is highly immunogenic [3]. At present, some progress has been made in immunotherapy for TNBC. Immunotherapy stimulates the immune response of TNBC patients through active immunity, such as cancer vaccines, or passive immunity, such as adoptive T cell therapy, tumor-specific antibodies, and immune checkpoint inhibitors [4]. A study has fused TNBC cells with peripheral blood monocyte-derived dendritic cells (DCs) to generate DC vaccines, which stimulate the proliferation of T lymphocytes and enhance the cytotoxic effect on breast cancer cells [5]. Emerging immune checkpoint inhibitors have received increasing attention in numerous TNBC study. TNBC patients with higher PD-L1 expression levels and more tumor-infiltrating lymphocytes (TILs) have higher immunogenicity, which plays a crucial part in regulating the immune response [6]. FDA has approved PD-L1 blockade combined with chemotherapy for patients with PD-L1-positive TNBC [7].

Increasing evidence suggests that immune gene expression and TIL may be prognostic for TNBC. Rody et al. [8] analyzed the RNA sequencing data of 579 TNBC patients and find that the expression of immune cell metagenes is closely related to prognosis. A study found that TNBC patients with higher TILs have better overall survival (OS) [9]. Yeong et al. [10] found that high PD-1+ cell infiltration significantly improved disease-free survival in TNBC patients. Similarly, PD-1, IFNG, and IFN signaling genes are positively correlated with the improvement of clinical outcomes of TNBC patients. These studies suggest that immune genes and TILs play an important role in TNBC. However, there are currently no immune-related genes that individually and visually predict OS and TILs for patients with TNBC.

In this study, we analyzed the RNA-seq and microarray data in The Cancer Genome Atlas (TCGA) and Gene Expression Omnibus (GEO) databases to comprehensively evaluate immune-related genes (IRGs) expression levels and predict prognosis and immune cell infiltration in TNBC patients. The cell functions involved in IRGs were also explored. These explorations are especially important for the individual assessment of the prognosis of TNBC patients and the discovery of targeted immunization methods.

## 2. Materials and Methods

### 2.1. Acquisition of Datasets and Patients' Information

UCSC Xena (https://xenabrowser.net/datapages/) [11] was used to download the RNA-seq data of breast cancer in the form of log_2_ (norm_count + 1) from TCGA database (http://www.tcga.org/), including 123 newly diagnosed PR-, ER-, and Her2-negative breast cancers and 13 normal tissues adjacent to tumor. At the same time, the clinical information of patients was obtained, involving age, gender, tumor invasion depth (T), lymph node metastasis (N), distant metastasis (M), TNM stage, survival time, and status. The 123 TNBC patients in TCGA database served as a training cohort, and the clinical characteristics are listed in Table S1. In addition, a microarray dataset and clinical characteristics from 107 TNBC cases obtained from the GSE58812 dataset [12] in the GEO database were used as a validation cohort.

### 2.2. Differential Analysis of Genes

The limma package in R (version 3.6.1) was used to select differential genes between 123 cases of primary TNBC and 13 normal tissues adjacent to tumor. A total of 20,530 genes were included into the differential analysis, setting the adjusted *p* value < 0.05, log_2_  | fold change | >1, and expression level > 0.2. And a total of 1076 genes with significant differences were finally determined. We focused on the role of IRGs in TNBC. The ImmPort database (https://www.immport.org/) [13] provides and updates 2498 IRGs for cancer research, all of which have been identified as being involved in the biological processes of immunity. Therefore, we downloaded the list of IRGs from the ImmPort database. Finally, 4.8% of IRGs were identified as those (a total of 119) differentially expressed genes and were included in subsequent analysis. Functional enrichment analysis of KEGG (Kyoto Encyclopedia of Genes and Genomes) and GO (Gene Ontology) was performed on 119 IRGs through the Database for Annotation, Visualization and Integrated Discovery (DAVID, https://david.ncifcrf.gov/) to explore potential molecular mechanisms.

### 2.3. Construction and Validation of Nomogram Model

The foreign and rms packages in R (version 3.6.1) were applied for establishing a nomogram model to analyze the role of IRGs in TNBC patients, which was described in detail in our previous study [14]. First, according to the nomogram, a point is given for each patient's IRG expression level. Then, a total risk score is obtained by gathering the given points of all the IRGs of the patients, which can predict OS. The concordance index (C-index) of 1000-sample bootstrap and the receiver operating characteristic (ROC) curve were used to evaluate the prognostic prediction ability of the nomogram model, and the judgment criterion was Area Under the Curve (AUC) or C‐index > 0.5. Model performance was assessed through both the internal and external calibration curve of 1000-sample bootstrap.

### 2.4. The Clinical Value of Risk Score

The risk score was divided into high or low based on the median value. Risk distribution, survival status, and IRG expression distribution of high- and low-risk TNBC were plotted by the heat map package in R (version 3.6.1). Survival and survminer packages were used to draw survival curves. We also explored the predictive value of risk score in TNBC for immune cell infiltration. The TIMER database (http://cistrome.org/TIMER/) [15] analyzed the TILs of 32 cancers in TCGA. Therefore, we obtained TIL abundances in TNBC patients from the TIMER and evaluated the correlation between the risk score and TILs.

### 2.5. Statistical Analysis

All statistical analysis was conducted in R (version 3.6.1, https://www.r-project.org/). Uni- and multivariate Cox regression analyses were used to screen prognostic variables. A log-rank test was used to compare the difference between survival curves. Two sets of quantitative data were compared by the Wilcoxon test. The correlation between the two sets of quantitative data was expressed by Spearman coefficient. A two-tailed *p* value < 0.05 was considered statistically significant.

## 3. Results

### 3.1. Identification of Differential IRGs

**Figure 1:**
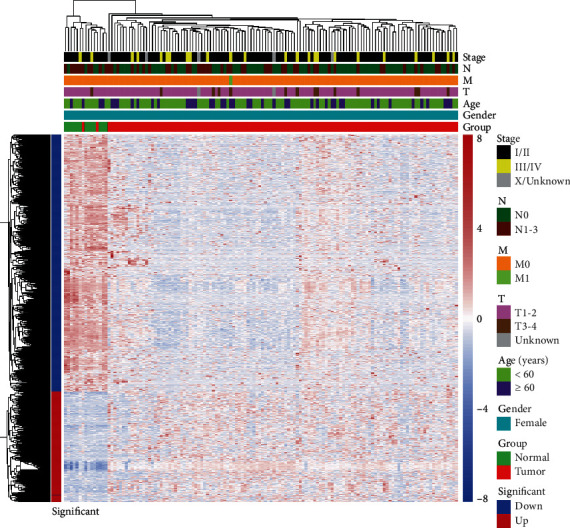
The heat map shows the differential genes between 123 cases of primary TNBC and 13 normal tissues adjacent to tumor. The color scale from green to red represented the difference in gene expression from low to high.

**Figure 2:**
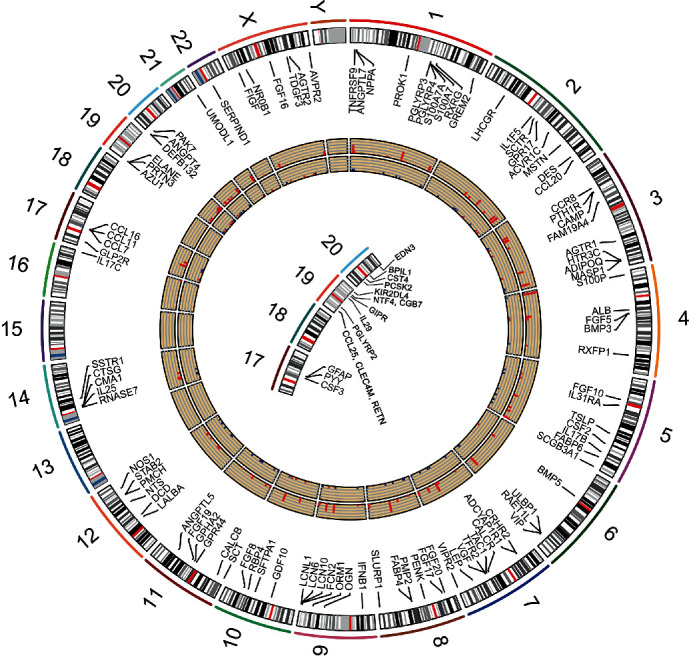
Differentially immune-related genes (IRGs) and their chromosomal locations. The number in the outermost circle is the name of the chromosome. The second circle is the positions of the genes in the chromosome. The black and white bars represent the chromosome bands, and the red bars represent the centromeres. The third circle is the gene names. The fourth circle is the average expression levels of the genes, and the bars from low to high represent the gene expression from low to high. The fifth circle is the fold change of genes. Blue represents fold change < −2, while red represents fold change > 2. In the center of the circle diagram is the positions of genes that cannot be fully displayed on the second circle.

**Figure 3:**
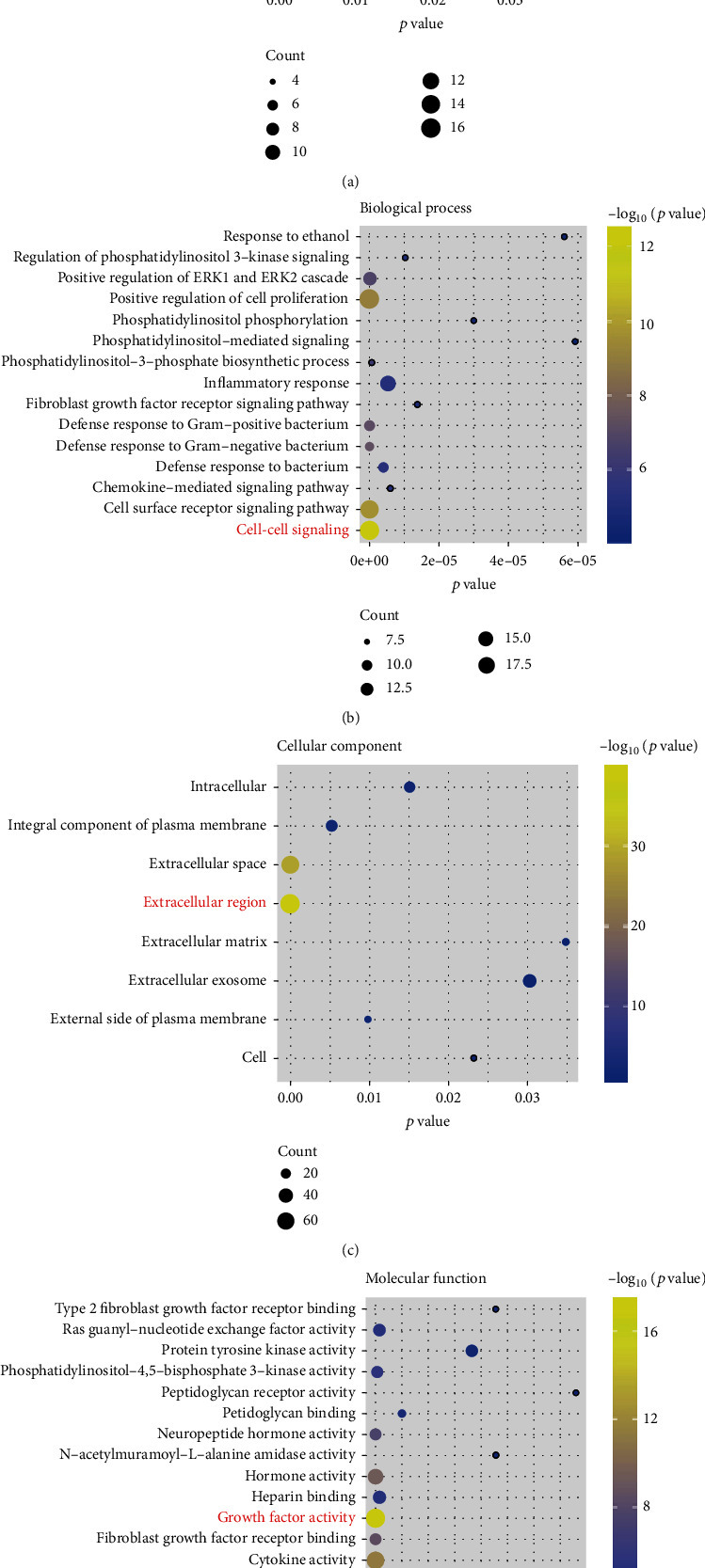
Functional enrichment of differential IRGs, and the top 15 of items are displayed. (a) KEGG pathway analysis; (b) biological process; (c) cellular component; (d) molecular function. The bubbles in the figures from small to large indicate that the number of enriched genes is from small to large. The color scale from blue to red indicates that the *p* value is from large to small.

Differential analysis found that a total of 1076 genes were differentially expressed in TNBC, including 323 high expression and 753 low expression genes (Figure 1). Among them, a total of 119 differential IRGs, including 36 high expression and 83 low expression IRGs, were found. Their positions on the chromosome are shown in Figure 2. The KEGG showed that the enrichment of differentially expressed IRGs was primarily in the “neuroactive ligand-receptor interaction” and “cytokine-cytokine receptor interaction” (Figure 3(a)). Biological processes, cellular components, and molecular functions are primarily enriched in “cell-cell signaling,” “extracellular region,” and “growth factor activity,” respectively (Figures 3(b)–3(d)). These findings suggested that cytokines and receptor pathways were most frequently implicated.

### 3.2. Identification of Prognosis-Related IRGs

**Figure 4:**
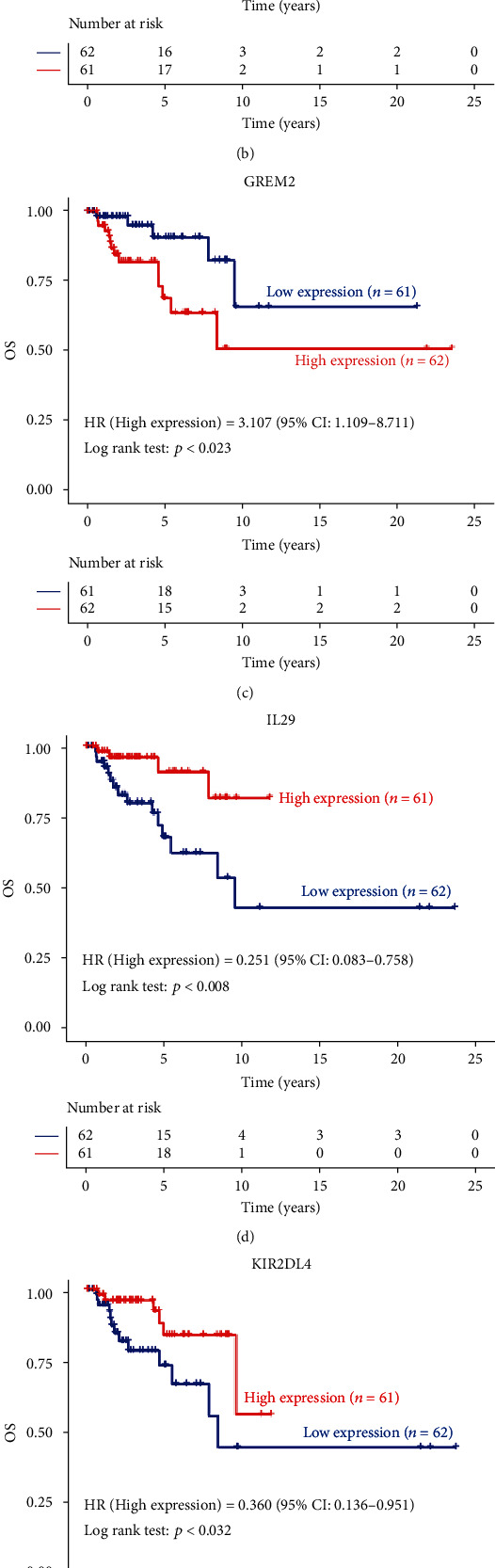
Survival curve analysis of prognostic IRGs. Survival analysis based on the expression levels of 119 IRGs shows that 6 genes are closely related to overall survival (OS).

Because prognostic molecular biomarkers are important for guiding treatment and disease monitoring, we focus on the impact of IRGs on the OS in TNBC. Prognostic analysis revealed that a total of 6 IRGs had significant impact on the OS of TNBC patients. Among them, high expression of C-C motif chemokine ligand 25 (CCL25), interleukin 29 (IL29), teratocarcinoma-derived growth factor 3 (TDGF3), and killer cell immunoglobulin like receptor, two Ig domains and long cytoplasmic tail 4 (KIR2DL4) predicted a favorable OS in TNBC patients (Figures 4(a), 4(d), 4(e), and 4(f)). Conversely, high expression of G protein-coupled receptor 44 (GPR44) and gremlin 2, DAN family BMP antagonist (GREM2) predicted a poor OS in TNBC patients (Figures 4(b) and 4(c)).

### 3.3. Establishment of Nomogram Model

**Figure 5:**
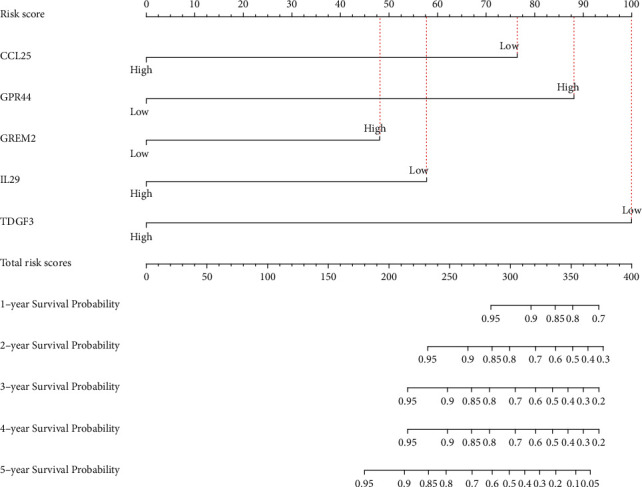
Construction of nomogram model with prognostic IRGs for predicting 1-, 2-, 3-, 4-, and 5-year OS in TNBC patients. A higher risk score meant a lower survival probability.

**Figure 6:**
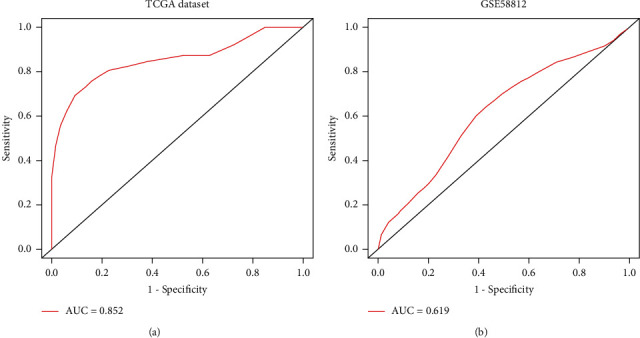
The ROC curves for the nomogram models. (a) ROC curve verified the nomogram model constructed by CCL25, IL29, TDGF3, GPR44, and GREM2. (b) ROC curve verified the nomogram model constructed by prognostic IRGs (CCL25, GPR44, GREM2, IL29, and TDGF3) in the dataset GSE58812 of the GEO database.

**Figure 7:**
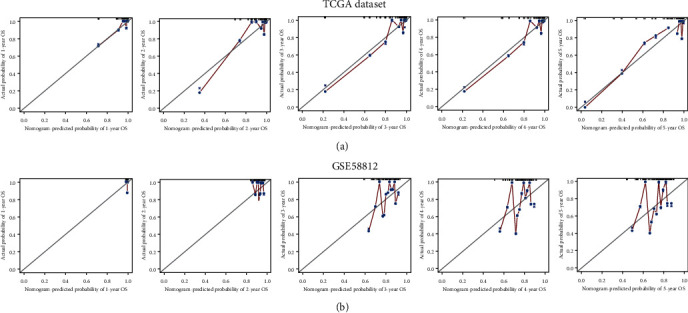
Internal and external calibration curve validation of the nomogram model. (a) Calibration curves of internal validation in TCGA database. (b) Calibration curves of external validation in the GSE58812 dataset.

CCL25, IL29, TDGF3, KIR2DL4, GPR44, and GREM2 obtained from the analysis of TCGA database were included into the establishment of a nomogram model. According to the expression levels of these IRGs, we got the total risk score of each individual, which could predict 1-, 2-, 3-, 4-, and 5-year OS (Figure S1). However, we could observe from Table S2 that the upregulated KIR2DL4 accounted for only 5 risk scores in the model. Compared with the risk scores of the other five IRGs, KIR2DL4 had a lower contribution to predicting the OS of TNBC patients, so we excluded KIR2DL4 in the establishment of the nomogram model. Finally, five prognosis-related IRGs including CCL25, IL29, TDGF3, GPR44, and GREM2 were included into the establishment of the nomogram model (Figure 5). To assess the predictive effect of the nomogram model on the OS of TNBC patients, we used the AUC of the ROC curve and the C-index of 1000-sample bootstrap for evaluation. When KIR2DL4 was not removed, the AUC was 0.839 and C-index was 0.878 (Figure S2). When KIR2DL4 was excluded, AUC was 0.852 and C-index was 0.879 (Figure 6(a)). Further external validation of the nomogram model with the GSE58812 dataset in the GEO database showed that the AUC of the ROC curve was 0.619 and the C-index is 0.615 (Figure 6(b)). We also used the calibration curves to further validate the nomogram model. The calibration curves for the training group showed a good consistency between the predicted and actual 1-, 2-, 3-, 4-, and 5-year OS of the nomogram model (Figure 7(a)). At the same time, the calibration curves in the validation group were also well identified (Figure 7(b)). Through internal and external verification, it was proved that the nomogram model in this study could conduct relatively accurate prediction of the OS of TNBC patients.

### 3.4. Risk Stratification

**Figure 8:**
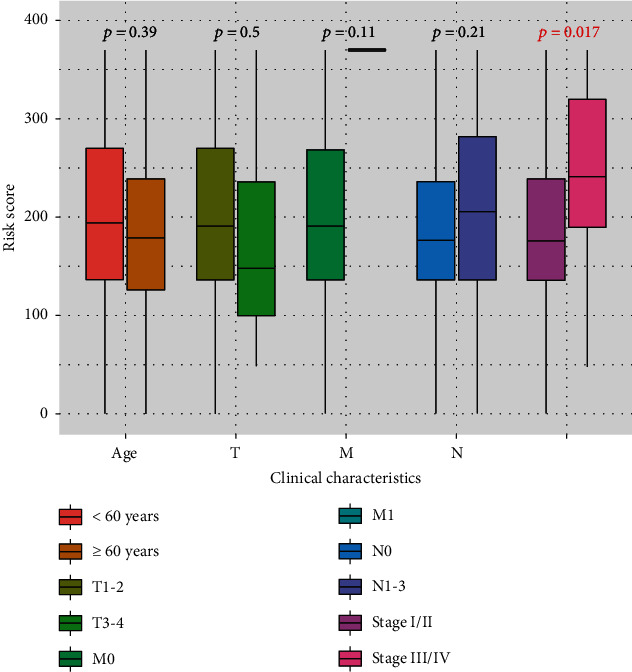
The distribution of risk scores in TNBC patients among different age, tumor invasion depth (T), distant metastasis (M), lymph node metastasis (N), and TNM stage.

**Figure 9:**
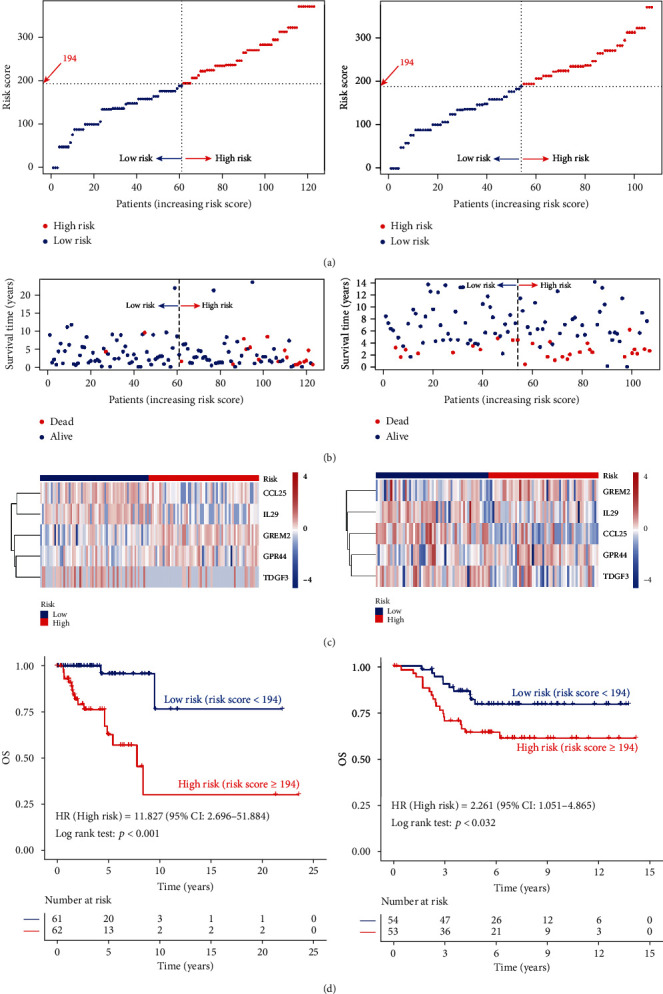
Risk stratification of TNBC patients by risk score. (a) Risk score, (b) survival time, and (c) gene expression distribution of low- and high-risk groups in TCGA dataset (left panel) and GSE58812 (right panel). (d) Plotting survival curves according to low- and high-risk scores in TCGA dataset (left panel) and GSE58812 (right panel).

In order to analyze the clinical value of the risk score, we compare the risk scores of different clinical information and found that compared with stage I/II TNBC patients with stage III/IV had higher risk scores (*p* = 0.017), and there was no significant difference between different ages, T, N, and M (*p* > 0.05) (Figure 8). After uni- and multivariate Cox regression analyses, we found that the risk score was an independent risk factor (HR = 1.019, 95% CI 1.012-1.027, *p* < 0.001, Table 1). The risk score was highly correlated with TNM stage, indicating that risk score could be used to construct risk stratification. TNBC patients can be defined as low-risk (risk score < 194) or high-risk (risk score ≥ 194) cohort based on the risk score (Figures 9(a)–9(d), left panel). As shown in Figure 9(d), left panel, the survival curve of low- and high-risk TNBC patients showed significant differences (*p* < 0.001). This finding could be validated by the external dataset GSE58812 (*p* = 0.032, Figures 9(a)–9(d), right panel).

### 3.5. Correlation between Risk Score and TILs

**Figure 10:**
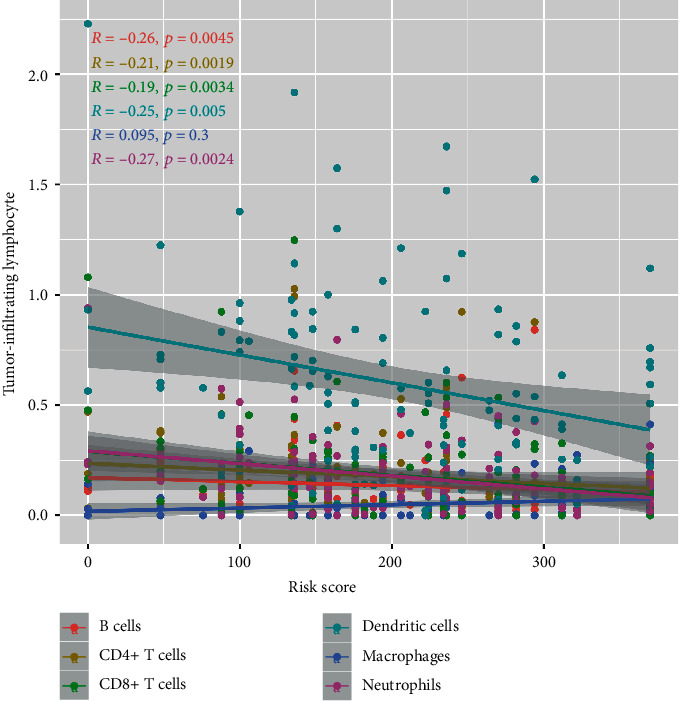
Spearman correlation between risk score and tumor-infiltrating lymphocytes (TILs).

In order to investigate whether the risk score reflected by immune genes could accurately assess the state of TNBC immune microenvironment, we performed the correlation analysis between risk score and TILs (Figure 10) and found that risk score was negatively related to B cells (*R* = −0.26, *p* = 0.005), CD4+ T cells (*R* = −0.21, *p* = 0.019), CD8+ T cells (*R* = −0.19, *p* = 0.034), dendritic cells (DCs) (*R* = −0.25, *p* = 0.005), and neutrophils (*R* = −0.27, *p* = 0.002). However, the risk score had no significant correlation with macrophages (*p* = 0.3).

## 4. Discussion

The clonal proliferation and metastasis characteristics of cancer cells depend on genome changes. We focus our research on changes in the immune genome to reveal its relationship with the immune microenvironment. In this study, we extracted differential IRGs from two large TNBC cohorts in the GEO and TCGA databases and analyzed the underlying immune mechanism. Pathway and GO analysis found that TNBC patients primarily function through interactions between cytokines and receptors. Various studies have showed that cytokines and receptors are involved in the growth, invasion, and metastasis of breast cancer, and corresponding immune inhibitors against cytokines and receptors have been applied to the treatment of breast cancer [16, 17]. In addition, fibroblast growth factor receptors (FGFRs) are highly expressed in TNBC patients, and inhibitors against FGFRs have been tested in clinical trials [18]. Bioinformatics analysis provided clues that changes in the immune system of TNBC patients were initiated through cytokine and receptor pathways, which laid the foundation for in-depth immune-related research.

The nomogram model has been widely applied to systematically assess the outcome of cancer patients [14, 19]. At the same time, IRGs can provide personalized immune signature to assess the prognosis of lung cancer patients [20]. The prognosis-related CCL25, IL29, TDGF3, GPR44, and GREM2 in IRGs were used to construct a nomogram model to evaluate its clinical value in TNBC patients. The nomogram model we constructed can individualize and visualize 1-, 2-, 3-, 4-, and 5-year OS for TNBC patients. Evidence suggests that blocking the CCR9/CCL25 axis can promote tumor progression and distant metastasis [21, 22]. IL29 appears to inhibit tumor growth in a variety of cancers [23]. TDGF3, also known as TDGF1P3 or CRIPTO3, is expressed in cancer [24]. Our study found that high expression of CCL25, IL29, and TDGF3 predicted a good prognosis in TNBC patients. Study has shown that GPR44 is involved in the release of cytokines from immune cells and the development of inflammation [25]. Silencing GREM2 can inhibit the JNK signaling pathway in gastric cancer, which inhibits tumor growth [26]. In this study, high expressions of GPR44 and GREM2 could predict adverse outcomes in TNBC patients. The establishment of risk stratification can provide a reference for clinicians to more rationally manage cancer patients and individualized treatment plans [27]. We conducted risk stratification for TNBC patients based on the risk score and found that patients with a risk score ≥ 194 belonged to a high-risk cohort, while patients with a risk score < 194 belonged to a low-risk cohort.

The risk score showed good clinical practicability. The risk score was an independent risk factor for TNBC patients and was positively related to TNM stage. In addition, the risk score was negatively correlated with B cells, CD4+ T cells, CD8+ T cells, dendritic cells, and neutrophils, which could reflect the level of TILs and provide a reference for clinicians to adjust the treatment plan. Studies indicate that a favorable prognosis is observed in TNBC patients with high B cells and CD4+ T cells [28, 29]. Furthermore, it has been reported that activated CD8+ T cells have been shown to kill cancer cells through various mechanisms [30]. Not surprisingly, less infiltration of CD8+ T cells into tumors in TNBC patients is often related to worse outcomes [31]. In addition, a study suggests that neutrophil infiltration is a favorable prognostic factor for colon cancer [32], which is consistent with our findings. As we know that DCs take a vital part in the tumor microenvironment, infiltration of activated DCs into tumors can improve the antitumor effect of immune cells [33]. In this study, high-risk patients might have the lower infiltration levels of B cells, CD4+ T cells, CD8+ T cells, dendritic cells, and neutrophils, which was associated with a poor OS.

The limitation of this study was that no clinical samples and corresponding clinical information were used to validate the nomogram model and risk stratification constructed by CCL25, IL29, TDGF3, GPR44, and GREM2. Additionally, the reliability of the results was still challenged because we also lacked validation in vitro and in vivo.

## 5. Conclusion

A nomogram model constructed by CCL25, IL29, TDGF3, GPR44, and GREM2 could predict the 1-, 2-, 3-, 4-, and 5-year OS of TNBC patients and perform risk stratification. The risk score derived from the nomogram model could also predict the level of immune cell infiltration in tumors. These findings provided a reference for personalized prognosis prediction in TNBC patients and might be potential immune biomarkers for designing novel therapy.

## Figures and Tables

**Table 1:** Uni- and multivariate Cox regression analyses of 123 TNBC patients in TCGA database.

Variables	Univariate Cox		Multivariate Cox	
	HR (95% CI)	*p* value	HR (95% CI)	*p* value
Risk score	1.022 (1.014-1.031)	<0.001^∗∗∗^	1.019 (1.012-1.027)	<0.001^∗∗∗^
Age (years)	1.004 (0.969-1.040)	0.842		
Tumor invasion depth				
T1-2	Reference			
T3-4	2.986 (0.976-9.140)	0.055		
Distant metastasis				
M0	Reference		Reference	
M1	54.325 (4.926-599.140)	0.001^∗∗^	3.099 (0.272-35.283)	0.362
Lymph node metastasis				
N0	Reference		Reference	
N1-3	3.785 (1.437-9.967)	0.007^∗∗^	1.778 (0.483-6.548)	0.387
Stage				
I/II	Reference		Reference	
III/IV	5.441 (2.113-14.012)	<0.001^∗∗∗^	4.273 (1.043-17.515)	0.044^∗^

HR: hazard ratio; CI: confidence interval; ^∗^*p* < 0.05; ^∗∗^*p* < 0.01; ^∗∗∗^*p* < 0.001.

## Data Availability

The data used and analyzed during the current study are available from UCSC Xena platform (https://xenabrowser.net/datapages/) and GEO database (https://www.ncbi.nlm.nih.gov/geo/). The data that support the findings of this study are available from the corresponding author upon reasonable request.

## Supplementary Materials

Supplementary 1 Figure S1: construction of a nomogram model with CCL25, IL29, TDGF3, KIR2DL4, GPR44, and GREM2 for predicting 1-, 2-, 3-, 4-, and 5-year OS in TNBC patients.

Supplementary 2 Figure S2: ROC curve verified the nomogram model constructed by CCL25, IL29, TDGF3, KIR2DL4, GPR44, and GREM2.

Supplementary 3 Table S1: the clinical information of TNBC patients.

Supplementary 4 Table S2: the risk scores for variables in nomograms.
