# Supplementary figures and images for: Nomogram Personalizes and Visualizes the Overall Survival of Patients with Triple-Negative Breast Cancer Based on the Immune Genome
Source: Biomed Res Int. 2020 Nov 24;2020:4029062. doi: 10.1155/2020/4029062 (PMC7709499; doi:10.1155/2020/4029062)

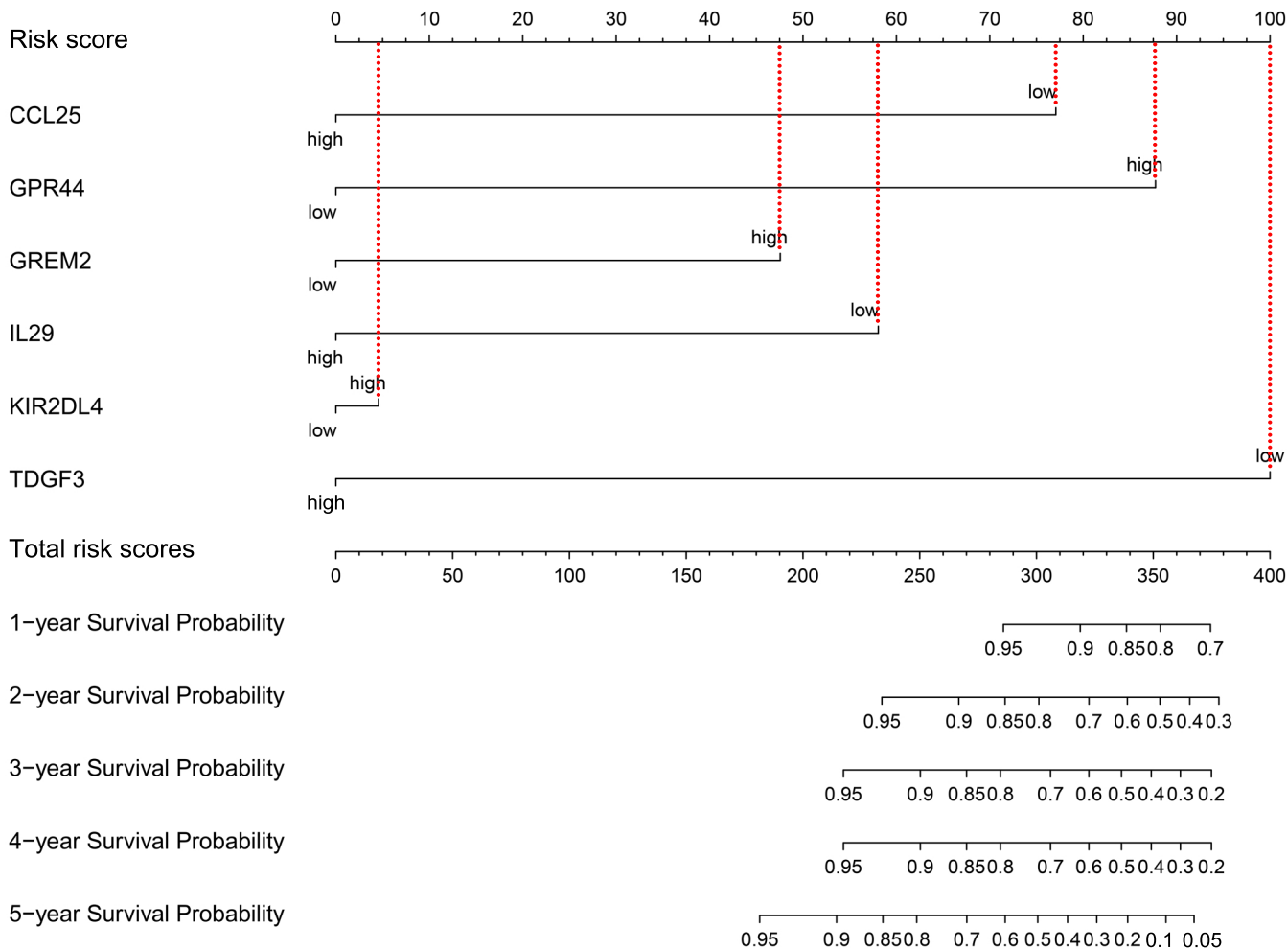

Supplement: Supplementary 1 — Figure S1: construction of a nomogram model with CCL25, IL29, TDGF3, KIR2DL4, GPR44, and GREM2 for predicting 1-, 2-, 3-, 4-, and 5-year OS in TNBC patients. [file 4029062.f1.pdf]

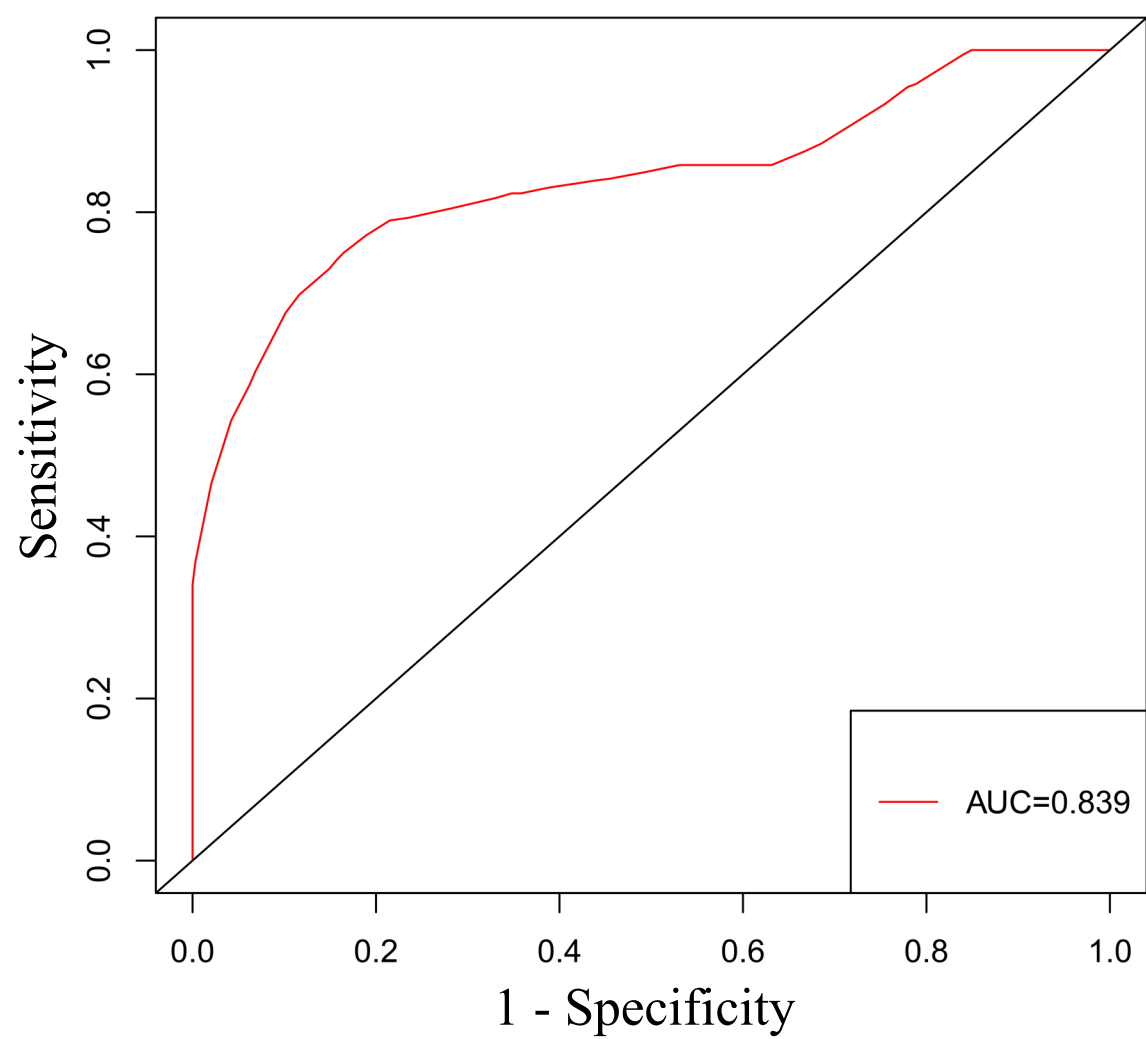

Supplement: Supplementary 2 — Figure S2: ROC curve verified the nomogram model constructed by CCL25, IL29, TDGF3, KIR2DL4, GPR44, and GREM2. [file 4029062.f2.pdf]
