# Supplementary material for: Nomogram Personalizes and Visualizes the Overall Survival of Patients with Triple-Negative Breast Cancer Based on the Immune Genome
Source: Biomed Res Int. 2020 Nov 24;2020:4029062. doi: 10.1155/2020/4029062 (PMC7709499; doi:10.1155/2020/4029062)
Supplement: Supplementary 3 — Table S1: the clinical information of TNBC patients. [file 4029062.f3.docx]

**Table S1.** Clinical information of TNBC patients.

| Variables | Training cohort  (TCGA) | Validation cohort  (GSE58812) |
| --- | --- | --- |
| Number | 123 | 107 |
| Age, years, n (%) |  |  |
| <60 | 83 (67.5) | 64 (59.8) |
| ≥60 | 40 (32.5) | 43 (40.2) |
| Gender, n (%) |  |  |
| Male | 0 (0.0) | - |
| Female | 123 (100.0) | - |
| Tumor invasion depth, n (%) |  |  |
| T1 | 26 (21.1) | - |
| T2 | 82 (66.7) | - |
| T3 | 11 (9.0) | - |
| T4 | 2 (1.6) | - |
| TX | 2 (1.6) |  |
| Lymph node metastasis, n (%) |  |  |
| N0 | 78 (63.4) | - |
| N1 | 30 (24.4) | - |
| N2 | 9 (7.3) | - |
| N3 | 6 (4.9) | - |
| Distant metastasis, n (%) |  |  |
| M0 | 122 (99.2) | - |
| M1 | 1 (0.8) | - |
| Stage, n (%) |  |  |
| I | 19 (15.5) | - |
| II | 77 (62.6) | - |
| III | 21 (17.1) | - |
| IV | 1 (0.8) | - |
| X | 3 (2.4) |  |
| Unknown | 2 (1.6) |  |
| OS, years, median  (Minimum, maximum) | 2.644 (0.014, 23.575) | 5.567 (0.055, 14.110) |
| Event (0 / 1), n | 104 / 19 | 78 / 29 |
| para‑non‑tumor tissue, n | 13 | - |
