# Supplementary material for: Nomogram Personalizes and Visualizes the Overall Survival of Patients with Triple-Negative Breast Cancer Based on the Immune Genome
Source: Biomed Res Int. 2020 Nov 24;2020:4029062. doi: 10.1155/2020/4029062 (PMC7709499; doi:10.1155/2020/4029062)
Supplement: Supplementary 4 — Table S2: the risk scores for variables in nomograms. [file 4029062.f4.doc]

**Table S2. Risk scores for variables in nomograms.**

| Variables | Risk score | | Survival time | Survival probability | Total risk scores | |
| --- | --- | --- | --- | --- | --- | --- |
|  | Before adjustment | After adjustment |  |  | Before adjustment | After adjustment |
| CCL25 |  |  | 1 year | 0.95 | 286 | 284 |
| low | 77 | 76 |  | 0.90 | 319 | 317 |
| high | 0 | 0 |  | 0.85 | 339 | 337 |
| GPR44 |  |  |  | 0.80 | 353 | 351 |
| low | 0 | 0 |  | 0.70 | 374 | 373 |
| high | 88 | 88 | 2 year | 0.95 | 234 | 232 |
| GREM2 |  |  |  | 0.90 | 267 | 265 |
| low | 0 | 0 |  | 0.85 | 287 | 285 |
| high | 48 | 48 |  | 0.80 | 301 | 299 |
| IL29 |  |  |  | 0.70 | 322 | 321 |
| low | 58 | 58 |  | 0.60 | 339 | 337 |
| high | 0 | 0 |  | 0.50 | 353 | 351 |
| KIR2DL4 |  |  |  | 0.40 | 366 | 364 |
| low | 0 | - |  | 0.30 | 378 | 377 |
| high | 5 | - | 3 year | 0.95 | 217 | 215 |
| TDGF3 |  |  |  | 0.90 | 250 | 248 |
| low | 100 | 100 |  | 0.85 | 270 | 268 |
| high | 0 | 0 |  | 0.80 | 285 | 283 |
|  |  |  |  | 0.70 | 306 | 304 |
|  |  |  |  | 0.60 | 322 | 321 |
|  |  |  |  | 0.50 | 336 | 335 |
|  |  |  |  | 0.40 | 349 | 347 |
|  |  |  |  | 0.30 | 362 | 360 |
|  |  |  |  | 0.20 | 375 | 373 |
|  |  |  | 4 year | 0.95 | 217 | 215 |
|  |  |  |  | 0.90 | 250 | 248 |
|  |  |  |  | 0.85 | 270 | 268 |
|  |  |  |  | 0.80 | 285 | 283 |
|  |  |  |  | 0.70 | 306 | 304 |
|  |  |  |  | 0.60 | 322 | 321 |
|  |  |  |  | 0.50 | 336 | 335 |
|  |  |  |  | 0.40 | 349 | 347 |
|  |  |  |  | 0.30 | 362 | 360 |
|  |  |  |  | 0.20 | 375 | 373 |
|  |  |  | 5 year | 0.95 | 181 | 180 |
|  |  |  |  | 0.90 | 214 | 213 |
|  |  |  |  | 0.85 | 234 | 233 |
|  |  |  |  | 0.80 | 249 | 247 |
|  |  |  |  | 0.70 | 270 | 269 |
|  |  |  |  | 0.60 | 287 | 285 |
|  |  |  |  | 0.50 | 301 | 299 |
|  |  |  |  | 0.40 | 313 | 312 |
|  |  |  |  | 0.30 | 326 | 324 |
|  |  |  |  | 0.20 | 339 | 338 |
|  |  |  |  | 0.10 | 355 | 354 |
|  |  |  |  | 0.05 | 367 | 366 |
